# Supportive care needs of adolescents and young adults 5 years after cancer: a qualitative study

**DOI:** 10.3389/fpsyg.2024.1268113

**Published:** 2024-04-30

**Authors:** Valentine Baudry, Magali Girodet, Mathilde Lochmann, Margaux Bottichio, Emilie Charton, Cécile Flahault, Anne-Sophie Baudry, Amandine Bertrand, Véronique Christophe

**Affiliations:** ^1^Département des Sciences Humaines et Sociales, Centre Léon Bérard, Lyon, France; ^2^UMR (Research Unit) Inserm 1052–CNRS (National Center of Scientific Research) 5286 Centre de Recherche en Cancérologie de Lyon, Université Claude Bernard Lyon 1, Lyon, France; ^3^U1290 Inserm RESHAPE: Research on Healthcare Performance, Université Claude Bernard Lyon 1, Lyon, France; ^4^Département de la Recherche Clinique et de l’Innovation, Centre Léon Bérard, Lyon, France; ^5^Laboratoire de Psychopathologie et Processus de Santé, Université Paris Cité, Paris, France; ^6^Pôle Cancérologie et Spécialités Médicales, Centre Hospitalier de Valenciennes, Valenciennes, France; ^7^Institut d’Hémato-Oncologie Pédiatrique, Lyon, France

**Keywords:** supportive care needs, adolescents and young adults (AYA), cancer, long-term follow-up, survivors

## Abstract

**Introduction:**

Adolescent and young adult (AYA) survivors who have been treated for cancer during childhood and adolescence are at great risk of the physical, psychological, and social consequences of cancer and its associated treatments. However, compliance with long-term follow-up is low. One possible explanation is that follow-up care fails to meet the expectations of AYA survivors. This study explored the specific supportive care needs of AYA survivors of childhood and adolescent cancer five years post-diagnosis.

**Methods:**

Semi-structured interviews were conducted with 15 AYA aged 15 to 25 years old. Thematic analyses were conducted to establish categories of supportive care needs and classify them as being met or unmet.

**Results:**

Participants reported between 2 and 20 specific needs (M = 11), including needs concerning fertility issues and reassurance regarding relapse (each mentioned by 67% of AYA), followed by the need for locomotor care, follow-up coordination and multidisciplinary care (60% of AYA for each). Participants also reported needs regarding social relationships, administration and finance, and academic and professional domains. Most (69%) of these needs were reportedly unmet, including need of information about cancer repercussions and follow-up, support in managing fatigue and sleep problems, psychological assistance, and support from peers.

**Discussion:**

The supportive care needs are still considerable and varied in AYA survivors of childhood and adolescent cancer 5 years post-diagnosis and are largely unmet. As unmet supportive care needs highlight the gap between available care in follow-up and the real needs of AYA survivors, a better understanding of their supportive care needs and unmet needs, thanks to systematic needs assessment, would enable long-term follow-up care to be adapted, thereby improving compliance and quality of life.

## 1 Introduction

Cancer affects 2,500 children and adolescents (0–19 years old) every year in France (INCa, 2015). Children and adolescents treated for a cancer have beneficiated from the evolution of therapeutics allowing the progressive increase in the 5-year survival rate reaching today more than 80% in developed countries (Ward et al., 2014; INCa, 2022). However, the growing number of survivors of childhood and adolescent cancer has made visible the number of long-term complications they often have to endure and that can make their after-cancer experience being challenging (Sadak et al., 2013). The physical consequences of cancer and its treatments on survivors that have been treated for cancer in late childhood or adolescence are diverse, frequent and potentially serious in the long term (Oeffinger et al., 2006; Blaauwbroek et al., 2007; Armstrong et al., 2014). Thus, the need for medical follow-up care is well established (Children’s Oncology Group, 2018), though some pediatric oncologists do not seem fully aware of guidelines or feel comfortable with survivors of 21 years old and older (Henderson et al., 2010). At the psychological level, childhood and adolescent cancer survivors have to deal with the negative impact of cancer and its treatments on numerous areas of their life. They are at increased risk of severe symptoms or a psychiatric diagnosis of depression, anxiety or psychotic disorder, even 5 years post-treatment or after (Hovén et al., 2020; Lee et al., 2023). Feelings of uncertainty, increased anxiety (Belpame et al., 2019; Conway Keller et al., 2020), ongoing distress (Raphael et al., 2019) are reported, and emotional disturbances, behavioral problems and drug misuse (Friend et al., 2018) are also more likely to appear in this population. Integrating cancer and adolescent identity into a cancer survivor identity can be complex and indicative of the level of acceptation of the experience of cancer (Stegenga and Macpherson, 2014; Darabos and Ford, 2020); some survivors report the impact of cancer on their functioning, their feeling of being different although trying to tend toward normality (Belpame et al., 2019). Previous work has shown their need to have access to psychological support, but these resources often seem to be lacking (Hendriks et al., 2020). Existing literature highlights an increased risk of experiencing difficulties at school, at work and financially: childhood survivors are more likely to present poorer performance at school, to be unemployed, to have a lower occupational position or to have less income than general population, especially for central nervous system tumor survivors (Mader et al., 2016; Frederiksen et al., 2019), although one can argue that the financial problematics and their seriousness vary depending on countries and health covering politics. Furthermore, the cognitive consequences of cancer treatments toxicity and periods of absence from school or work can create issues not only in the academic and professional domain but also in developing and/or maintaining friendships and romantic relations (Evan and Zeltzer, 2006; Martinez-Santos et al., 2021). However, despite recommendations concerning the need for multidisciplinary individualized follow-up, only 20% of pediatric survivors regularly attend specialized consultations, even when they suffer severe late effects (Rebholz et al., 2011). This lack of attendance could be explained by their supportive care needs not being met or only partially.

The French National Cancer Institute defines supportive care as “all the care and support needed by patients throughout the course of the disease together with specific onco-hematological treatments, when they are needed.” Supportive care is part of patient care and aims to preserve quality of life in its physical, psychological and social dimensions. It is intended to address the numerous challenges and difficulties that can occur during cancer: pain, fatigue, nutritional and digestive problems, but also social difficulties, psychological distress and end-of-life questions (Que sont les soins de support? - Soins de support, 2020). Supportive care is therefore key to creating a better quality of comprehensive and personalized care, not only for patients but also for their carers. The degree to which the supportive care needs of cancer patients are met seems to have an impact on their quality of life (So et al., 2014; Sodergren et al., 2019). From a healthcare perspective, needs refer to problems that may or may not ultimately be solved. Supportive care needs can be unmet when supportive care is not received or is insufficient (Lambert et al., 2012). Unmet needs seem to be impacted by socioeconomic factors (Shakeel et al., 2020), clinical factors (Fiszer et al., 2014; Okediji et al., 2017; Shakeel et al., 2020), emotional competence, anxiety and depression symptoms (Baudry et al., 2018), fear of recurrence (Ellegaard et al., 2017) and post-traumatic growth (Nik Jaafar et al., 2022). In adolescent and young adult (AYA) patients, supportive care is needed once their treatment has finished (Sender et al., 2019), and illness, health-related and psychological factors are known to have an impact on unmet needs for AYA survivors of childhood cancer (Lamore et al., 2020). Zhang et al. (2023) found a negative relationship between self-rated health and unmet needs in survivors of AYA cancer (Zhang et al., 2023). Unmet needs can also be a sign of adjustment difficulties and impaired quality of life for AYA survivors of childhood and adolescent cancer (Quinn et al., 2015; Galán et al., 2018; Nicklin et al., 2019). Furthermore, compliance with follow-up care may be associated with unmet information needs (Knighting et al., 2020). A lack of compliance with follow-up care may indicate that some supportive care needs are met too late or insufficiently. This lack of compliance may be multifactorial (e.g., unmet needs of psychological support about fear of recurrence, distance of care facilities from home, lack of information about the importance of follow-up or existing services, motivational aspects, etc.; Ernst et al., 2022), yet numerous issues about the real needs of AYA survivors of childhood and adolescent cancer in the long term have received little attention. The investigation of unmet supportive care needs may serve to demonstrate inadequacies in the support provided by health professionals (Baudry et al., 2019); this exploration therefore seems paramount to fill gaps and adapt existing follow-up care and programs. Several studies have been conducted on AYA cancer survivors’ needs, but the specific needs and unmet needs of survivors that have been treated a few years ago during late childhood, and that are now in the sensible period of adolescence and young adulthood, have not been enough explored. We also wished to conduct an exploratory study, without any restriction regarding the type or domain of needs.

If the supportive care needs of AYA survivors of childhood and adolescence cancer could be clearly identified, their attendance at consultations should increase. We hypothesize that (un)met needs are impacted by a variety of individual and cancer factors in AYA and that they have an impact on their adjustment to cancer post-treatment and on compliance with follow-up care. Based on the observation that AYA survivors’ supportive care needs are not well enough known yet, the main objective of this qualitative study is to explore specific supportive care needs of AYA survivors of childhood and adolescent cancer up to six months after the end of the oncologic surveillance, i.e., 5 years post-diagnosis.

## 2 Materials and methods

### 2.1 Design

This study is part of a larger project exploring the supportive care needs of survivors from 6 to 30 years old at the time of the study (divided into three age subgroups), and their parents. This article focuses on qualitative data regarding cancer survivors from 15 to 25 years old at the time of the study (i.e., 10 to 20 years old at diagnostic). The study was single-center, prospective, observational, cross-sectional and qualitative. It was conducted in a pediatric institute and an adult unit of a Comprehensive Cancer Centre in France. All survivors aged 15 to 25 years old, diagnosed 5 years before, who had completed their oncological treatments were invited to participate. The range of this age group was based on the AYA program at our center which follows French standards of care, although international definitions of AYAs’ age range varies greatly across regions and healthcare systems (e.g., the National Cancer Institute defines AYA as individuals between 15 and 39 years old). The choice of the time since diagnosis allowed us to interview survivors at the end of their oncological surveillance, i.e., when they were declared in remission. For greater clarity, we will be using the term “AYA survivors” to mention our population of interest in the following pages, referring to survivors that have been treated either during late childhood or adolescence and that are now adolescents or young adults (i.e., from 15 to 25 years old at the time of the study).

The study was conducted in accordance with the Declaration of Helsinki, the French regulations on clinical trials, and with the authorization of the Ethics Committee of Lille University (project number: 2021-469-S90).

### 2.2 Variables and data collection

Supportive care needs were explored through recorded individual semi-structured interviews. Demographic information was assessed using a self-questionnaire.

### 2.3 Inclusion criteria

Survivors could participate if they (1) were aged 15 to 25 years old at the time of the study, (2) were former patients of the center, (3) were treated for a solid tumor or a lymphoma, (4) were in remission and had finished their standard oncological surveillance since 1 to 6 months (i.e., were at 5 years since diagnosis), (5) were able to read and write French, and (6) declared that they were not opposed to the study.

### 2.4 Procedure

Eligible survivors were screened from the hospital database. The study was presented to the AYA survivors (and their parents, if minors) either by their oncologist during the last surveillance consultation, or by a researcher on the phone. The study was explained to them and a written information notice was given or sent to them. They were invited to ask any questions they might have and could spend time deciding whether or not they wanted to participate. If non-opposition was obtained, an appointment was then scheduled during which the survivor participated in a semi-structured interview with a researcher, completed a questionnaire and underwent a medical consultation to explore treatment sequalae and to provide guidance regarding medical follow-up with a specialized physician.

### 2.5 Interviews

The interviews were conducted by a psychology researcher and audio-recorded with the participant’s agreement. During the interviews, participants were asked to answer the questions spontaneously. The first explorative question was the following: “As a cancer survivor, could you tell me about your current needs and expectations in terms of care or any other type of help?” (see Supplementary material). Then the interviewer used dunning to probe the themes further, if necessary. In the second part of the interview, more oriented questions were asked about specific types of needs in three categories: physical needs, information needs and psychological, social and administrative needs, based on relevant theory in the literature (Bibby et al., 2017; Galán et al., 2018). This two-stage interview method (first “exploratory open” then “exploratory directive”) sought to explore what emerged spontaneously and then to reveal still undisclosed needs.

### 2.6 Analyses

Sample size was not calculated *a priori*, as no statistical analysis was performed on the main criterion that was assessed qualitatively. Recruitment stopped when data reached saturation, i.e., when new data is redundant and no new theme emerges (Saunders et al., 2018).

Descriptive analyses were conducted on the pseudonymized transcribed interviews using thematic analysis (Braun and Clarke, 2006). The analysis was performed on Nvivo^®^ software (release 1.6.1, January 2022). The texts were read by two researchers (VB & ML), then screened iteratively to generate an analytical grid organized in themes and subthemes. The coding took place individually and the researchers met regularly to discuss the grid relevance and possible questions that emerged. A double quotation was conducted on 20% of the interviews and potential discrepancies were discussed with the other researchers until a consensus was reached. Quantitative variables were described using mean (standard deviation) and median (minimum-maximum). Qualitative variables were described by absolute and relative frequencies.

Needs were coded as such if participants expressed clear lack of help, support or specific care, or their desire to have access to any support, care or service. Needs were then categorized as met, i.e., participants expressed a need that is resolved, either through offered supportive care, or by any other way (e.g., need that has faded with time), or unmet, i.e., participants expressed an unresolved need. Percentages of met and unmet needs refer to the number of occurrences found.

## 3 Results

Of 35 survivors contacted, 15 agreed to participate in the study (42.9%). Participants’ demographical and clinical characteristics are shown in Table 1. Reasons for refusal were: living too far away from the cancer center (*n* = 5, 25.0%), lack of time (*n* = 4, 20.0%), not wanting to come back to the cancer center (*n* = 3, 15.0%), contact issues (*n* = 3, 15.0%), lack of interest (*n* = 1, 5.0%) or the cancer experience still being too painful (*n* = 1, 5.0%). Three survivors (15.0%) gave no reason for their refusal. Interviews lasted 29.7 min on average, ranging from 12 to 59 min.

**TABLE 1:** Participants’ characteristics (*n* = 15).

	*n*	%
**Age at diagnosis (years)**		
Mean (standard deviation)	15 (1.73)	
Median (range)	14 (12–18)	
**Age at inclusion (years)**		
Mean (standard deviation)	20 (1.89)	
Median (range)	19 (16–23)	
**Gender**		
Female	8	66.7
Male	4	33.3
**Type of tumor**		
Central nervous system	2	13.3
Lymphoma	6	40.0
Soft-tissue sarcoma	2	13.3
Bone	4	26.7
Germ cell and gonadal	1	6.7
**Chemotherapy**		
No	1	6.7
Yes	14	93.3
**Immunotherapy/targeted therapy**		
No	15	100.0
Yes	0	0.0
**Radiotherapy**		
No	6	40.0
Yes	9	60.0
**Surgery**		
No	6	42.9
Yes	8	57.1
**Education level**		
<high school diploma	4	33.3
=high school diploma	6	50.0
>high school diploma	2	16.7

### 3.1 Number and type of needs

**FIGURE 1:**
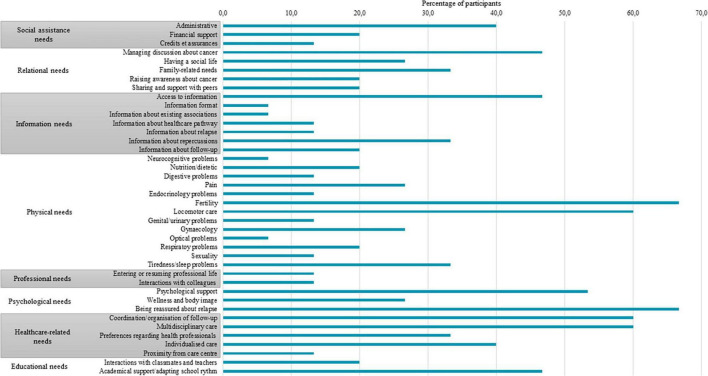
Proportion of participants expressing needs for each subtheme.

Eight themes of needs and 40 related subthemes were identified. The main themes concerned physical, psychological, healthcare-related, relational, informational, social assistance, academic and professional needs. Themes and subthemes are presented in Figure 1. Survivors expressed an average of 11 needs (median: 10; range: 2–20).

#### 3.1.1 Physical needs

All the participants (100%) mentioned need for support or follow-up in the physical domain. Fertility need was one of the most expressed needs in this domain (expressed by 10 participants out of 15, i.e., 66.7%), sometimes seen as a future need.

“*Hum*… *maybe a fertility specialist. For later, when I’ll want to have a child. Yes, I think I’ll need this health professional*” (*20-year-old female participant*).

Locomotor needs regarding rehabilitation or adapted physical activities were expressed by nine participants (60.0%).

“*Well, you should know that I had a reaction to one of the chemotherapies and I ended up really disabled in the legs and the hands. I almost couldn’t walk, hold a pen and so on. And I still can’t write. My hands, I still have a lot of problems. And I’m not particularly followed up on this*” (*19-year-old female participant*).

Physical needs also concerned tiredness and sleep disturbances (5/15, 33.3%), pain (4/15, 26.7%), gynecologic issues (4/15, 26.7%), respiratory problems (3/15, 20.0%), nutrition or dietetic (3/15, 20.0%), digestive problems (2/15, 13.3%), genital and urinary problems (2/15, 13.3%), endocrinology (2/15, 13.3%), sexuality (2/15, 13.3%), neurocognitive problems (1/15, 6.7%) and optical problems (1/15, 6.7%).

“*So it’s really weird, I’m in good shape but I’m really tired. For example, I can’t take naps. Although I’m K.O. But I can’t take naps. I don’t know why.*

*I: Ok. So about that, you would need support?*

“*P: Yes*” (*18-year-old female participant*)”.

“*What’s more, it’s true that it worries me and yes, I think it’s good to have follow-up on that. But it’s true no one talks about it [sexuality]*” (*18-year-old female participant*).

#### 3.1.2 Psychological needs

Psychological needs were expressed by 13 participants (86.7%). Participants mostly expressed the need to be reassured by the medical team regarding the fear of relapse (10 participants out of 15, 66.7%).

“*But I know it can come from everywhere. And that often it comes back, that’s the worst of it. And since I always attract disasters (laughs). So yes, that I*… *it’s true it would be nice if they could reassure us a bit about that. But it’s true that they finish that session too soon, I think. You’ve got five years of remission and then that’s it. You go on with your life. But it’s true that afterward many people relapse, and they’re surprised*” (*18-year-old female participant*).

They also mentioned the need to be supported by a mental health professional in order to emotionally process the experience of cancer and the period after treatment (8/15, 53.3%).

“*I was followed up very much, even in the beginning I was really followed up because I had lots and lots of problems. During, a little bit less, but because I didn’t need it. And after, not at all! And I think it’s a shame to have dropped away like that. It could have been just a little follow-up, I don’t say we need long-term follow-up. But it could be better I think*” (*21-year-old male participant*).

“*I didn’t really manage at the time (light laugh). It lasted a long time this period, when I wasn’t feeling OK. I’d say it lasted a good year, a year and a half. And it’s afterward I decided to go to see a psychologist, to talk about a lot of things, especially about my cancer. And afterward I began to be better. And then here I am (light laugh)*” (*19-year-old female participant*).

Some of them (4/15, 26.7%) expressed needs regarding wellness or accepting body image, through cosmetic and wellness care or plastic surgery, e.g., to hide scars.

“*I think maybe like wellbeing. Such as massages or stuff like that. Because personally I know we get used to pain, suffering, having another technique on our body could help us. Yes, I think like massage and stuff like that*” (*18-year-old female participant*).

#### 3.1.3 Needs related to healthcare

Thirteen (86.7%) participants expressed needs related to healthcare. In particular, they expressed needs regarding follow-up coordination and planning (9/15, 60.0%), i.e., being guided in managing follow-up care, finding health professionals or improving communication between those involved in healthcare.

“*Every time they gave me the plastic surgeon’s number, but it was me who had to contact him. These are things you don’t necessarily want to*… *So I would have liked*… *Well, this is my way of seeing it, me if I’m not forced in the medical domain, it’s a bit of a struggle. At the time, if the doctor had told me: this neurosurgeon exists, get an appointment next week. Well, it’s silly, but maybe that the relationship, yes, maybe create a system where we are accompanied and where everyone can refer to each other*…” (*19-year-old female participant*).

Many (9/15, 60.0%) mentioned the need for a multidisciplinary follow-up (including the need for alternative therapies), possibly as a one-stop shop.

Six (40.0%) mentioned the need for individualized follow-up care: for the professional to be proactive, to understand their specific needs and to adapt to them.

“*I: Do you need opportunities to meet members of the medical, paramedical, or care-coordination team to discuss and share things after treatment?*

*P: Well, yes and no, but in fact I find it useless because I feel that nobody, yes, nobody here ever gets to*… *well, to what I really want*” (*18-year-old female participant*).

Lastly, participants also expressed preferences regarding the choice of health professional for follow-up care (e.g., someone who is an expert in oncology, who knows one’s disease history, who is empathic/involved, etc.; 5/15, 33.3%) or regarding the geographical proximity of follow-up (2/15, 13.3%).

“*As soon as I had a problem—the problem is, now every problem is caused by what happened to me, of course: concentration problems, vision problems, all of that is linked to my illness actually—so necessarily I went by them [the oncology team] because I knew they were in the best position and that they would understand me better; whereas going to see someone, re-explain everything to them, who perhaps would have missed a step that I would have explained inadequately or not at all. Since they know everything about me, they know what I’ve been through, they’ve seen what care I didn’t have, etc. So it was going to be much better to go through them*” (*21-year-old male participant*).

#### 3.1.4 Relational needs

Relational needs were expressed by 11 participants (73.3%). Participants mentioned the need for support about how to deal with the issue of cancer in social situations (e.g., answering questions, managing others’ reactions, etc.; 7 participants out of 15, 46.7%), the need to return to a satisfactory social life and the importance of social bonds (4/15, 26.7%), or the need for sharing and support with peers affected by cancer (3/15, 20.0%).

“*But it does me good, really, the outings, the events, etc., that*… *it, it’s really helpful. Because we all need the social stuff and*… *well it’s good*” (*18-year-old female participant*).

Five (33.3%) participants expressed needs relating to the family domain: support in managing family affairs, in improving family communication or the need for support for another member of the family.

“*Maybe to discuss with someone fully, all the family together. It could have been something rather good, yes*” (*20-year-old male participant*).

Some also mentioned the importance of raising awareness about cancer or disablement in society (3/15, 20.0%), like this 15-year-old girl:

“*Well, I think it’s important to run prevention campaigns to show people that everyone can be concerned; for example, that you shouldn’t make fun and if it happens to you one day, you shouldn’t give up, because once you’ve recovered, you realize all you’ve been through and you can be proud of yourself*.”

#### 3.1.5 Information needs

The need for information, expressed by nine participants (60.0%), concerned cancer repercussions (5/15, 33.3%), follow-up (3/15, 20.0%), relapsing cancer (2/15; 13.3%), information about their oncological healthcare pathway (2/15; 13.3%) or the need to receive information about existing support or associations (expressed by one participant, 6.7%).

“*It’s true that I know now that I have to have follow-up after my remission. And it’s true I’m a little confused about that. So what I understand it’s that*… *That I have a medical visit afterward that will be of use. So that makes me feel better. It’s just that I was worried of being a bit lost.*

*I: Yes. So, at the end of the surveillance phase?*

*P: Yes, that’s it*” (*19-year-old female participant*).

Concerning relapse, several participants expressed the difficulty of distinguishing what can be considered ‘normal’ symptoms and signs of potential recurrence.

“*And there’s also what I explained to you with the relapses, information about treatment, how*… *when to worry, when not to worry*” (*19-year-old male participant*).

This domain also refers to needs relating to accessing information (7/15, 46.7%) or information format (1/15, 6.7%), like this young woman expressing a satisfied need:

“*I: You feel you had enough information?* *P: Yes well, really a lot, before, during and after. So frankly, one’s really stubborn if they say they haven’t had it!*” (*22-year-old female participant*).

#### 3.1.6 Social assistance needs

Social assistance needs were expressed by eight participants out of 15 (53.3%). Six (40.0%) mentioned the need for follow-up from a social worker or for support in procedures concerning various forms of administrative assistance: help getting the disabled worker status, help managing the administrative load while their parents used to take care of it, etc.

“*So yes, if I could get administrative support. Like I said, with the disability card, disabled worker status, can I, I don’t know, for example, will I be able to declare my taxes with my work-study program or not? How about housing, how will this work? It’s true that it would be nice to have guidance on this, yes*” (*21-year-old male participant*).

Some participants (2/15, 13.3%) also mentioned the need for financial support, especially to access healthcare specialists or services not covered by French social security. Two (13.3%) expressed needs regarding credit and insurance.

#### 3.1.7 Educational needs

Seven participants (46.7%) expressed needs relating to school life. For six (40.0%), this concerned adaptation of school rhythm or academic support: being guided and supported in teaching, especially following periods of absence from school.

“…*we adjusted; so generally, I was going half-day, sometimes less. As soon as I felt that I was tired, I told them and I could go home, there wasn’t any problem at all. In college, they’re flexible about it, I could leave and come back whenever I wanted; that way I could keep in touch, follow classes a bit without killing myself with it. So, from that perspective, it was really adapted*” (*18-year-old male participant*).

Four participants (26.7%) also mentioned needs relating to interaction with classmates or teachers, expressing the importance of making others aware in order to avoid stigmatization.

#### 3.1.8 Professional needs

Three participants (20.0%) expressed the need for support or guidance in the professional field. Two (13.3%) expressed the need for professional integration, guidance with one’s professional project or adaptation of working time. Two (13.3%) expressed needs regarding interaction with colleagues and hierarchy, like this 21-year-old male participant, expressing his fear of talking about his cancer to his superior:

“*Personally, I know that to avoid problems and get my work placement, I didn’t say anything. So I work like an ordinary person, even though I had major problems. So I told my boss I was going to have frequent appointments because I have a serious illness, but I don’t dare talk about it, I don’t want to shoot myself in the foot. Whereas if someone helps us, it could be helpful*.”

### 3.2 Met and unmet needs

**FIGURE 2:**
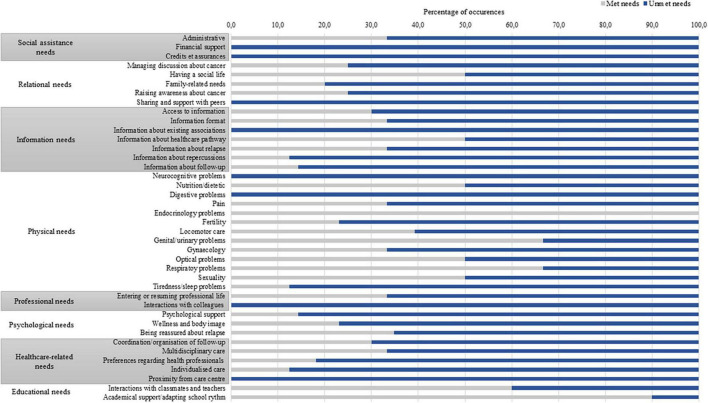
Proportion of met and unmet needs for each subtheme.

Of the needs expressed by the participants, 69.2% were unmet. The proportions of met and unmet needs for each subtheme are presented in Figure 2. Furthermore, all expressed at least one unmet need. The domains in which needs were the most frequently unmet concerned social assistance needs (81.8%), professional needs (80.0%) and information needs (78.8%). The only domain in which needs were mostly met was educational needs (only 20.0% of unmet needs). Concerning subthemes, some needs were exclusively or almost exclusively unmet, as the need for sharing and support with peers (100.0% unmet), the need for support about fatigue and sleep problems (87.5% unmet), the need for information about repercussions of cancer (87.5% unmet) and follow-up (85.7% unmet), the need for psychological support (85.7% unmet) and the need for individualized follow-up care (87.5% unmet).

### 3.3 Needs expressed during the “exploratory open” vs. “exploratory directive” parts

Forty-five percent of expressed needs emerged in the exploratory open part of the interview. Some subthemes exclusively or almost exclusively appeared in the exploratory directive part. For example, need for multidisciplinary care and needs concerning fatigue and sleep disturbances were expressed only in the exploratory directive part, when these needs were prompted based on supportive care needs, as described in the literature. Fertility needs (92.3%) and the need for assistance in managing discussions about cancer with others (91.7%) emerged in the exploratory directive part, when the participants were prompted. The needs expressed in the exploratory directive part were more unmet than those expressed in the exploratory open part (72.7 vs. 65.4%). Conversely, the need to be geographically near a care center and the need to have a social life were mostly expressed in the exploratory open part (respectively, 100.0 and 83.3% of these expressed needs).

## 4 Discussion

This study explored the supportive care needs of AYA survivors that have been treated for cancer during late childhood and adolescence, 5 years following diagnosis, one to six months after the end of their surveillance. Results show that supportive care needs are diverse in AYA survivors and that they are largely unmet. Eight domains of supportive care needs were highlighted in the sample: physical, psychological, healthcare, relational, informational, social assistance, educational and professional needs. Among the subthemes, the most reported needs concerned fertility and being reassured about relapse, followed by locomotor care, coordination and organization of follow-up and multidisciplinary care. The needs for psychological support and social support were also largely expressed.

These results partly confirm those of other studies. AYA are known to have specific needs: need for age-adapted information, notably about fertility (Benedict et al., 2021), for adapted services and structures or the need for emotional support (Bibby et al., 2017). Bradford et al. (2020) found that 62% of AYA survivors express the need for care and information about after treatment (Bradford et al., 2020). Similarly, the survivors in our study all reported the need for physical care and half of them expressed the need to have access to information, notably about repercussions and follow-up. As in our study, the need for psychosocial support is mentioned in numerous studies about AYA survivors’ needs (Keats et al., 2019; Hendriks et al., 2020; Sender et al., 2020). The need to be supported by a mental health professional was one of the most expressed and unmet needs, which shows that this type of support is still lacking even five years after diagnosis. However, caution is required when comparing studies. For example, the definition of the AYA age group varies between authors and different regions in the world, and can often extend up to 39 years old. Our age group definition was based on the organization of care in French hospitals and in our cancer center, which has a dedicated AYA program. What’s more, literature can sometimes be confusing between survivors that had had a cancer while they were AYA, or survivors that have been treated for cancer during childhood or early adolescence and are now AYA, as their experience and needs may be very different (Patterson et al., 2015). Moreover, some studies of AYA after cancer do not mention the time since diagnosis or since treatment, or they include very different time scales, yet supportive care needs may be evolutive throughout survivorship, i.e., AYA just finishing treatment may have very different needs than those whose treatment was completed more than 15 years before.

The need to be reassured regarding a potential relapse is not commonly reported in the AYA survivors literature, although it is in line with numerous articles highlighting the fear of recurrence in AYA survivors (Yang et al., 2019; Lea et al., 2020; Mikrut et al., 2020), and evidence of this need in adults (Balfe et al., 2016; Beesley et al., 2018). It can be linked to the feeling of abandonment by healthcare teams that some cancer survivors experience at the end of their treatment (Conway Keller et al., 2020). This need has no easy solution, as AYA survivors may want to be reassured yet still value the honest information provided by their oncologist (Gibson et al., 2005). There is evidence that adolescent survivors are at elevated risk of emotional vulnerability like anxiety and post-traumatic stress symptoms (McDonnell et al., 2017), so practitioners should take this eventuality into account. However, AYA survivors may need more support and information about remission, the surveillance period and which symptoms may be considered as normal as opposed to those that may predict a recurrence, as some mentioned in their interviews. This would enable them to regain a sense of control over the post-treatment phase and consequently to decrease their feeling of fear about recurrence and related needs. It also seems important to provide support or counseling regarding the ways in which the issue of cancer can be discussed with others, as this can have an impact on the social relationships of AYA in a period where social life is crucial for them.

A third of the participants expressed needs regarding their family, which can also be considered as a scarce result, although it was reported by one other study (Barrett et al., 2020). Children and adolescents confronted with cancer are often supported by their parents and more broadly by their family members. Most of our subjects were aware of the repercussions of cancer not only on themselves but also on their family, and they variably expressed the need for support for their parents, siblings, and the whole family. This highlights the importance of offering support for carers and family members, as well as whole family support, even once treatment has ended.

Some needs were mostly unmet, which could testify to an overall lack of awareness of these issues by the healthcare system. The need for psychological support was still expressed five years after the initial diagnosis. There may be a delay between the diagnosis and the onset of psychological disturbances (Dauchy et al., 2013). The absence of psychological support, its unavailability or its inadequacy (Barrett et al., 2020) may be one manifestation among others of the lack of a systematically continuous care pathway or the difficulty to find a health professional once a patient has been discharged from hospital. Similarly, AYA survivors need to be able to meet and share with peers who also have had cancer, and they need to have access to information tailored to their needs, such as advice on sleeping disturbances and fatigue. Despite having seen their oncologist on a regular basis, these needs still exist and should be catered for. This raises the question of the extent of physicians’ knowledge and awareness of the needs of AYA survivors (Henderson et al., 2010), as well as their own needs and difficulties regarding the provision of follow-up (Michel et al., 2017). On the other hand, some AYA survivors may not be able to fully express their concerns and questions, perhaps owing to the fear of bothering their oncologist, as mentioned by some of the participants in our sample, as well as in the literature (Wakefield et al., 2013).

Although interventions have been developed to improve compliance with follow-up, they appear to be too scarce or insufficiently validated (Zabih et al., 2019). Few of the existing interventions target the psychological wellbeing of AYA (Raj et al., 2018), even though this is a major challenge. The transition between pediatric and adult follow-up may be insufficiently facilitated or accompanied (Tonorezos et al., 2018; Otth et al., 2021), which may lead to a breakdown in compliance with follow-up. The inclusion of the specific issues of AYA into follow-up still needs to be achieved (Barnett et al., 2016). To our knowledge, there is no comprehensive needs assessment scale for AYA survivors of childhood or adolescent cancer in either French or English; the questionnaires used in studies focusing on this population are either not specific to need assessment but still include one or two needs items (e.g., McClellan et al., 2013), are constructed specifically for one study (Gianinazzi et al., 2014; Vetsch et al., 2017; Hovén et al., 2018) or are not specific to this age group but are used on this purpose nonetheless (Sender et al., 2020).

The fact that our participants expressed some needs exclusively or almost exclusively during the second “exploratory directive” part of the interview can be interpreted in several ways. The first “exploratory open” part allowed us to collect needs expressed freely and spontaneously, without restricting ourselves only to data available in the literature. However, the positive responses to the prompts in the exploratory directive part do not mean that these needs do not exist. AYA are not always conscious of their own needs and may need help in expressing them. Health professionals should always investigate needs in depth and offer suggestions and advice. Some issues may be sensitive or taboo like sexuality, or not of immediate importance such as the question of fertility. Professionals may need to take the lead on mentioning these issues. AYA survivors of childhood or adolescent cancer may also be unaware of the lasting symptoms associated with cancer and its treatments like fatigue and sleep disturbances, yet they may become accustomed to coping with them on a daily basis (INCa, 2018). Furthermore, some survivors may not report problems for which they think there is no solution. In this regard, our two-stage process may have circumvented this problem to some extent.

### 4.1 Strengths and limitations

This study has several limitations. First, more than half of the survivors we contacted decided not to participate, a rate that appears high. Those who took part may have done so because they have more supportive care needs than those who did not. On the other hand, the latter may have more needs but were too disappointed by follow-up care to participate. In addition, several non-participants refused to participate because of the distance of the center from their home or because they did not want to come back to the hospital. This may also partly explain the lack of compliance with long-term follow-up. Second, the study was cross-sectional, which limits the possibility of drawing conclusions about the evolution of supportive care needs after treatment. Third, it was single-center, and approaches to supportive care during treatments may be different in this hospital from those adopted in another which can have an impact on supportive care needs after treatment. The qualitative methodology allowed us to explore in depth the supportive care needs of our sample, but our results cannot be generalized to the AYA survivor population. As adolescence and young adulthood is a period of rapid development and changes, we are aware that there may be great disparities in the expression of needs within our sample. However, a strength of our study is the homogeneity between participants regarding the time since diagnosis. Finally, our results could have been taken further by taking greater account of the school/professional/financial context of participants (e.g., by collecting data on job occupations or income) and by considering the participants’ use of supportive care needs, and the impact it has had on the expressed needs and their satisfaction regarding these needs.

During the delicate period of adolescence and young adulthood, cancer survivors may experience difficulties expressing their needs, either because they have other preoccupations, because they find it difficult to talk readily about themselves, or because they are uneasy about asking for help (Wakefield et al., 2013). Therefore, we may have missed some important issues. On the other hand, while our two-stage interview methodology has not been validated to our knowledge in AYA survivors, it may have allowed them to express themselves more easily when prompted on specific subjects.

## 5 Conclusion

Overall, our results contribute to the literature about the supportive care needs of AYA survivors of late childhood and adolescent cancer post-treatment. They point to trends for improving and adapting long-term follow-up care, notably in offering and facilitating access to long-term physical, psychological and social services, in guiding AYA survivors in their healthcare follow-up after the end of surveillance, providing them more tailored and specific information and helping them navigate school, social or professional life in a way that empower them and allow them to become independent actors of their health. Though the participants of our study had the opportunity of being treated in a hospital where a continuum exists between children and adults units, the question of their feeling of belonging and the non-specialization of units or flexibility of offered care needs to be raised for this population. The study also highlights the difficulty and the importance of evaluating supportive care needs over time, even during and after regular oncological follow-up. The sole assessment of somatic problems is insufficient, as supportive care needs may exist even in the absence of major physical problems. The subjective experience and autonomy of survivors is of paramount importance, especially in this special age group. In addition, the supportive care needs of each person differ depending on their history, their perceptions and their experience, so it is important to adapt care to each individual. Furthermore, AYA survivors’ needs are all the more specific as they have to manage a number of complex transitions and becoming independent from their parents, who until now could have dealt with a large number of cancer-related issues. The healthcare pathway of AYA cancer survivors being made of disruptions (e.g., end of treatment, end of surveillance, transition between pediatric and adult units), regular screening is important. The challenges of long-term follow-up should be clearly explained to these survivors on an ongoing basis, since their developmental status is constantly evolving (Dauchy et al., 2013). For these reasons, the systematic evaluation of the supportive care needs of AYA after treatment seems crucial. By assessing their risk factors, expectations, comprehension and needs, it becomes possible to propose early adapted interventions and to decrease the rate of complications and limit their individual and societal impact. Health professionals also need to be trained in the specific needs of AYA survivors and to be helped in finding ways to solve them. Creating multidisciplinary teams specialized in AYA survivors posttreatment, possibly in specialized centers outside of the hospital walls, would be particularly relevant given that AYA value AYA-specific services (Jones et al., 2017), the diverse nature of their supportive care needs and unmet needs, and the challenge to involve them more actively in their follow-up (Sadak et al., 2013). This would positively impact survivors’ compliance with follow-up, as well as their quality of life.

Future longitudinal, multicentre, quantitative research is needed to extend these findings and catch the dynamic aspect of supportive care needs in the AYA survivors’ population. Future development of this particular work will be to validate the interactions that exist between supportive care needs and various outcomes like quality of life and psychological distress. This will hopefully lead to improving the post-treatment experience of cancer survivors.

## Data availability statement

The datasets presented in this article are not readily available because access to data cannot be granted immediately since the dataset is still being analyzed in view of further publications. Requests to access the datasets should be directed to VC, veronique.christophe@lyon.unicancer.fr or AB amandine.bertrand@ihope.fr.

## Ethics statement

The studies involving humans were approved by the Ethics Committee of Lille University. The studies were conducted in accordance with the local legislation and institutional requirements. Written informed consent for participation was not required from the participants or the participants’ legal guardians/next of kin in accordance with the national legislation and institutional requirements.

## Author contributions

VB: Conceptualization, Formal analysis, Investigation, Methodology, Writing – original draft, Writing – review & editing. MG: Conceptualization, Formal analysis, Funding acquisition, Investigation, Methodology, Supervision, Writing – review & editing. ML: Formal analysis, Investigation, Methodology, Writing – review & editing. MB: Formal analysis, Writing – review & editing. EC: Formal analysis, Methodology, Writing – review & editing. CF: Conceptualization, Writing – review & editing. A-SB: Conceptualization, Writing – review & editing. AB: Conceptualization, Formal analysis, Funding acquisition, Investigation, Methodology, Supervision, Writing – review & editing. VC: Conceptualization, Formal analysis, Funding acquisition, Methodology, Supervision, Writing – review & editing.
