# Supplementary material for: Supportive care needs of adolescents and young adults 5 years after cancer: a qualitative study
Source: Front Psychol. 2024 Apr 30;15:1268113. doi: 10.3389/fpsyg.2024.1268113 (PMC11091414; doi:10.3389/fpsyg.2024.1268113)
Supplement: Supplementary file 1 [file Data_Sheet_1.pdf]

Supplementary

<u>Exploratory open part</u>

Opening question: As a cancer survivor, could you tell me about your current needs and expectations in terms of care or any other type of help?

Dunning:

- What could you tell, or advise, to someone at the end of their cancer treatment to help them prepare for the post-cancer period?
- What have you missed, or are you currently missing, in terms of care or other kinds of help?

<u>Exploratory directive part</u>

Supportive care needs relating to the physical experience

Do you need support or care relating to:

1. pain
2. tiredness or lack of energy
3. your weight or nutritional problems
4. digestive disorders
5. respiratory disorders
6. genito-urinary problems: burning, urinary problems (painful or uncomfortable urination, very frequent urination, etc.), genital pain, irritation, etc.
7. motor disorders: partial or total motor impairment, particularly of the lower and upper limbs
8. sleeping problems
9. sexuality or romantic relationships
10. the ability to have children
11. not being able to do things as before cancer
12. Do you have any other support or care needs related to your physical experience?

Supportive care needs related to post-cancer care and information

Do you need information or explanations about post-cancer care?

Do you need opportunities to meet one or more members of the medical, paramedical or care co-ordination team, to discuss and exchange information about after-cancer?

Do you need support and/or advice from health professionals (psychologist, social worker, specialist nurse, socio-aesthetician, physiotherapist, dietician, gynaecologist, sexologist, fertility specialist, etc.)?

Do you need a specialised structure bringing together health professionals to provide diversified post-cancer care (psychologist, social worker, specialist nurse, socio-aesthetician, physiotherapist, dietician, sexologist, fertility specialist, etc.)?

Do you need the contact details of healthcare professionals for post-cancer care (psychologist, social worker, specialist nurse, socio-aesthetician, physiotherapist, dietician, sexologist, fertility specialist, etc.)?

Supportive care needs related to psychological experience, family and social life, and professional and administrative life

Do you need support or care related to your psychological experience (anxiety-depressive disorders, anorexia, identity and existential issues such as life goals, return to a "normal" life, body image/self-esteem, sexual maturity, etc.)?

Do you have any expectations or needs related to:

1. worries about cancer relapse
2. worries about daily life or the future after cancer
3. the remission of your cancer
4. returning to a social life after cancer
5. family life after cancer (communication, functioning, understanding, concerns, support, etc.)
6. dealing with the subject of cancer in social situations (raising the subject with those around you, answering questions at work or school, etc.)
7. returning to a satisfactory school or work life
8. administrative procedures
9. social assistance and related services
10. insurance

Would you like to add something before the end of the interview?
